# Expression of Matrix Metalloproteinase-2/9 and Tissue Inhibitor of Metalloproteinase-1/2 as Predictive Factors in Oropharyngeal Squamous Cell Carcinoma

**Published:** 2019-05

**Authors:** Pawel-Krzysztof Burduk, Piotr Sawicki, Lukasz Szylberg, Magdalena Bodnar, Andrzej Marszalek

**Affiliations:** 1 *Department of Otolaryngology,Oncology and Oral and Maxillofacial Surgery, Faculty of Health Sciences, Nicolaus Copernicus University in Toruń, Poland.*; 2 *Private ENT Practice, Bydgoszcz, Poland.*; 3 *Department of Clinical Pathomorphology Collegium Medicum, Faculty of Medicine, Nicolaus Copernicus University in Toruń, Poland.*; 4 *Department of Oncologic Pathology, Poznan University of Medical Sciences and Greater Poland Oncology Center, Poznan, Poland.*

**Keywords:** Metalloproteinases, Oropharyngeal cancer, Survival, Tissue inhibitors

## Abstract

**Introduction:**

Metalloproteinases and their tissue inhibitors play an important role in the metastases formation. A multistage process of carcinogenesis requires the involvement of numerous enzymes and compounds that facilitate the expansion of tumor cells. The formation of metastases depends on both metalloproteinases and tissue inhibitors activation leading to the activation of neoangiogenesis. The changes of the expression in stromal and tumor proteins could be prognostic factors in patients with oropharyngeal squamous cell carcinoma.

**Materials and Methods:**

This study was conducted on a total of 34 patients with squamous cell carcinoma of the oropharynx divided into 2 groups, including 20 patients with neck metastasis and 14 patients without lymph node metastasis. Immunohistochemistry was performed with a standard protocol.

**Results:**

The results of the present analysis indicated a higher expression of metalloproteinases 2 in the stroma than in tumor with increasing tumor grade. The dynamics of changes in the expression of metalloproteinases showed the increase in metalloproteinases 2 and the decrease in metalloproteinases 9 depending on the tumor size. Dynamics of changes in the expression of tissue inhibitor 1 in the tumor stroma significantly increased with the tumor stage. In the assessment of nodal staging from N0 to N3, the expression of tissue inhibitor 1 and 2 were higher in the tumor tissues. The increase of metalloproteinases 2, tissue inhibitor 1 in the tumor, and metalloproteinases 9 in the stroma were characterized by a reduction in the odds ratio of patient’s survival.

**Conclusion:**

The complex evaluation of the expression of metalloproteinases and tissue inhibitors may be used for the prognosis of the patient’s survival.

## Introduction

Oropharyngeal squamous cell carcinoma is an increasingly frequent clinical problem. In recent years the number of diagnosed cases has significantly increased, particularly among younger patients. Therefore, it seems reasonable to search for new biochemical markers with high sensitivity and specificity, which would be useful in routine diagnostics.

Matrix metalloproteinases (MMPs) belong to the group of proteolytic endopeptidases. The MMP-dependent mechanisms are involved in both tumor development and formation of metastases to lymph nodes in the head and neck squamous cell carcinoma (HNSCC) (1,2). Metalloproteinases from the gelatinase family, including matrix metalloproteinase-2 (MMP-2) (gelatinase A, collagenase-4) and matrix metalloproteinase-9 (MMP-9) (gelatinase B, belong to a very important group from the perspective of carcinogenic mechanisms of oropharyngeal and tonsillar squamous cell carcinomas.

A multistage process of carcinogenesis requires the involvement of numerous enzymes and compounds that facilitate the expansion of tumor cells to other organs. Overexpression of MMP-2 and MMP-9 induces the degradation of the extracellular matrix. One of the mechanisms of metastases formation dependent on both metalloproteinases is the secondary activation of vascular endothelial growth factor and transforming growth factor beta leading to the activation of neoangiogenesis (1,2). On the other hand, the influence of tissue inhibitors of metalloproteinases (TIMPs), which are endogenous inhibitors of MMPs on tumor cells, both dependent and independent of the extracellular matrix, is considered (2,3). The increased expression of tissue inhibitor of metalloproteinase-1 (TIMP-1) is observed in patients with HNSCC. It is associated with faster tumor progression and shorter survival. 

However, the results in the literature are not consistent regarding this issue (4-6). The tissue inhibitor of metalloproteinase-2 (TIMP-2) does not show any organ specificity. In addition, TIMP-2 can inhibit progression and angiogenesis. The present study was carried out to analyze the changes in the expression of stromal and tumor proteins as prognostic factors in patients diagnosed with oropharyngeal squamous cell carcinoma.

## Materials and Methods

This study was conducted on a total of 34 patients with squamous cell carcinoma of the oropharynx divided into 2 groups, including 20 patients with neck metastasis and 14 patients without lymph node metastasis. The study was performed on 28 men (mean age: 56.5 years) and 8 women (mean age: 54.9 years). The study was approved with a decision number of KB 589/2011 by local Ethics Commission. Immunohistochemistry analysis was performed with the standard protocol. To establish immunohistochemical procedures, the authors have made several positive control reactions on a model tissue (Referring to The Human Protein Atlas, http:// www. proteinatlas. org). The negative control reactions were performed on additional tissue using the primary antibody of 1% bovine serum albumin diluted in phosphate buffered saline.

Mouse monoclonal antibody was used against MMP-2 (HPA001939; Sigma–Aldrich, Poznan, Poland; dilution 1:100), MMP-9 (ab58803; Abcam, Cambridge, UK; clone: 56-2A4; dilution 1:100), TIMP-1 (M7293; Dako, Glostrup, Denmark; clone: VT7, dilution 1:50), and TIMP-2 (ab1828; Abcam; clone 3A4, dilution 1:50). Epitopes were unmasked by Epitope Retrieval Solution high-pH (Dako, United States) and then slides were incubated with primary antibody overnight at 4C°.

The detection of interested antibody complex was performed with EnVision Anti-Mouse/Rabbit HRP Labeled Polymer (Dako, United States). Then antigens complex was localized according to the presence of the brown product of reaction using diaminobenzidine as a chromogen (Dako, United States). The results were observed and recorded at x20 magnification in the light microscope ECLIPSE E800 (Nikon Instruments Europe, Amsterdam, Netherlands) and the antigen expression was counted using morphometric principles by graphic program ImageJ (version 1.46a) to analyze the microphotography. The algorithmic program was used for the estimation of the expression intensity and the number of positive tissue area. The Microsoft Excel and R programs (http://www.r-project.org) were used for the statistical analyses. The Shapiro-Wilk test was used as a nonparametric test to assess the normality of the data. The Mann–Whitney U test was utilized to analyze the correlation between lymph node involvements and survival period in the studied groups. Moreover, the analysis of other parameters was performed using the Wilcoxon and Kruskal-Wallis tests. P-value less than 0.05 was considered statistically significant.

## Results

The analysis of the expression of selected metalloproteinases and tissue inhibitors was performed a) in cells of oropharyngeal squamous cell carcinoma and b) in tumor stroma. Additionally, in statistical analyses, MMP-2 in tumor-stroma (T-S), MMP-9 (T-S), TIMP-1 (T-S), and TIMP-2 (T-S) variables were introduced in order to determine the differences in the expression of metalloproteinases and their inhibitors between the tumor and stroma.

The conducted analysis demonstrated a statistically significant higher expression of MMP-2P in the stroma in comparison to the expression of MMP-2N in the tonsillar tumor (P<0.0001). In the case of MMP-9, statistically significant lower expression values were observed in stromal tissue (P=0.0445) in comparison to those in tumor tissue. There was also a statistically significant correlation for the variable MMP-9 (T-S) (P=0.0002). 

No significant difference was observed in the expression of TIMP-1, either in tumor or stroma, although in tumor tissue the expression was higher. The expression of TIMP-2 was similar, and no statistical significance was noticed between tumor and stroma while the expression level was higher in tumor tissue. In both cases, the expression level of TIMP-1 and TIMP-2 as a difference in variables (T-S) was statistically significant (Table.1, P<0.0001). 

**Table 1:** Metalloproteinases and tissue inhibitors expression in tumor and stroma tissue

**Parameter**	$\bar{X}$	**Standard deviation**	**Minimum**	**Median**	**Maximum**	**Coefficient of variation**	**P-value (Shapiro-Wilk test)**	**P ** ^(t/W)^
MMP-2T	53.04	19.99	14.62	51.14	106.7	37.68	0.6496	
MMP-2S	59.5	7.37	50.05	58.18	93.23	12.39	<0.0001	
MMP-2 (T-S)	-6.46	16.58	-42.8	-1.5	17.63	-256.71	0.0246	0.1239^ W^
MMP-9T	63.41	11.2	44.05	62.65	92.13	17.66	0.5562	
MMP-9S	56.98	8.59	43.44	55.56	78.12	15.08	0.0445	
MMP-9 (T-S)	6.43	8.76	-13.69	7.38	22.54	136.29	0.6782	0.0002 ^t^
TIMP-1T	103.17	8.39	84.72	104	118.44	8.13	0.8352	
TIMP-1S	90.27	7.23	78.71	89.01	102.19	8.01	0.0513	
TIMP-1 (T-S)	12.9	5.56	2.59	12.76	24.09	43.1	0.4358	<0.0001^t^
TIMP-2T	89.26	7.61	70.84	89.56	108.72	8.53	0.4749	
TIMP-2S	80.03	6.78	59.36	81.51	94.04	8.47	0.1370	
TIMP-2 (T-S)	9.23	5.36	-6.24	8.31	21.66	58.06	0.4935	<0.0001^t^

T: Tumor, S: Stroma ,t: Student's t-test,W: Wilcoxon test  ,MMP-2: Matrix metalloproteinase-2

MMP-9: Matrix metalloproteinase-9, TIMP-1: Tissue inhibitor of metalloproteinase-1

Statistically significant differences in the expression of MMP-2N, MMP-9N, and MMP-2 variables were demonstrated depending on the presence of metastases in cervical lymph nodes. In all cases, these values were lower in patients with metastases (P=0.0045, P=0.0038, and P=0.0168). Mean values of MMP-2 expression in tumor for all degrees of organ staging (T2-T4) were lower in comparison to the expression assessed in the stroma. 

The size of the tumor increased with the decrease of TIMP-1 (T-S). The impact of other inhibitors and metalloproteinases on tumor size was insignificant. Furthermore, it was demonstrated that MMP-2N, MMP-2 (T-S), and MMP-9N variables affected the presence of tumor in the lymph nodes. The lower level of the above-mentioned characteristics accompanied the occurrence of tumor in the lymph nodes (Table.2). 

**Table 2:** Expression of metalloproteinases and tissue inhibitors, as well as T/N staging

**Parameter**	**Value**	**Tumor size**			**P-value**	**Lymph node metastasis**		**P-value**
		**T2**	**T3**	**T4**		**N0**	**N1-3**	
MMP-2T	Mean	44.50	61.82	51.03	0.1565^ A^	64.10	44.90	0.0045 ^t^
	Coefficient of variation	34.61	34.03	39.23		24.18	42.94	
MMP-2S	Mean	56.61	61.80	59.35	0.2718 ^KW^	60.41	58.83	0.8422 ^U^
	Coefficient of variation	6.52	18.74	6.21		17.04	7.40	
MMP-2 (T-S)	Mean	-12.11	0.01	-8.31	0.1799 ^KW^	3.69	-13.93	0.0038 ^U^
	Coefficient of variation	-129.10	83893.99	-233.17		191.63	-127.08	
MMP-9T	Mean	67.16	67.29	58.22	0.0691 ^A^	68.72	59.50	0.0168 ^t^
	Coefficient of variation	11.66	20.35	15.47		17.42	15.16	
MMP-9S	Mean	60.99	58.75	53.30	0.1896 ^KW^	59.20	55.35	0.1719^ U^
	Coefficient of variation	16.01	16.64	10.22		14.26	15.45	
MMP-9 (T-S)	Mean	6.16	8.54	4.92	0.6036^ A^	9.52	4.15	0.0817 ^t^
	Coefficient of variation	151.83	110.54	166.30		93.46	196.30	
TIMP-1T	Mean	107.20	103.35	100.73	0.2239 ^A^	101.97	104.05	0.4905 ^t^
	Coefficient of variation	4.31	10.66	7.16		10.07	6.59	
TIMP-1S	Mean	90.19	91.20	89.59	0.8648 ^A^	90.86	89.84	0.6940^ t^
	Coefficient of variation	7.15	8.63	8.45		8.86	7.52	
TIMP-1 (T-S)	Mean	17.01	12.15	11.14	0.0454 ^A^	11.11	14.22	0.1143 ^t^
	Coefficient of variation	18.50	49.13	48.48		53.46	35.28	
TIMP-2T	Mean	89.98	90.35	88.01	0.7258 ^A^	89.26	89.27	0.9995 ^t^
	Coefficient of variation	5.58	10.10	8.94		10.15	7.41	
TIMP-2S	Mean	77.30	81.98	80.07	0.3435 ^A^	81.27	79.13	0.4211 ^t^
	Coefficient of variation	6.60	8.84	8.91		10.82	6.17	
TIMP-2 (T-S)	Mean	12.67	8.37	7.94	0.1091^ A^	8.00	10.14	0.2630^ t^
	Coefficient of variation	27.02	71.22	65.85		42.90	62.77	

N: Nodes,T: Tumor, S: Stroma , A: Analysis of variance, t: Student's t-test, KW: Kruskal-Wallis test U: Mann–Whitney U test, MMP-2: Matrix metalloproteinase-2 , MMP-9: Matrix metalloproteinase-9 , TIMP-1: Tissue inhibitor of metalloproteinase-1

The results of one-way analysis of variance and the Kruskal-Wallis test showed statistical difference in the level of MMP-2N and MMP-9N metalloproteinases depending on the involvement degree of the cervical lymph nodes (N). Significant differences were observed regarding the MMP-2 expression in the tumor, and its level decreased with the increase in the stage of metastases in the lymph nodes (P=0.015). A similar significant correlation was noticed in the case of MMP-9 in the tumor (P=0.0403). The introduced MMP-2 (T-S) variable was characterized by a more negative difference of metalloproteinase expression for N3 feature in comparison to the lower regional stage and the group of patients without metastases to lymph nodes. Moreover, significant differences in the TIMP-2 (T-S) were observed. However, it exhibited a variable tendency depending on the metastases size (Table.3, P=0.0085). 

**Table 3:** Expression of metalloproteinases and tissue inhibitors, as well as N staging

		**Lymph node metastasis**			**P-value**
		**N0**	**N1+2**	**N3**	
MMP-2T	Mean	64.10	46.83	40.72	0.0150 A
	Coefficient of variation	24.18	38.77	55.82	
MMP-2S	Mean	60.41	59.17	58.12	0.8080 ^KW^
	Coefficient of variation	17.04	7.16	8.49	
MMP-2 (T-S)	Mean	3.69	-12.33	-17.40	0.0120^ KW^
	Coefficient of variation	191.63	-140.28	-113.39	
MMP-9T	Mean	68.72	60.96	56.33	0.0403 ^A^
	Coefficient of variation	17.42	13.14	19.55	
MMP-9S	Mean	59.20	56.93	51.92	0.0709 ^KW^
	Coefficient of variation	14.26	11.77	22.38	
MMP-9 (T-S)	Mean	9.52	4.03	4.41	0.2244 ^A^
	Coefficient of variation	93.46	232.24	121.07	
TIMP-1T	Mean	101.97	104.66	102.74	0.7174^ A^
	Coefficient of variation	10.07	6.77	6.58	
TIMP-1S	Mean	90.86	90.48	88.44	0.7935 ^A^
	Coefficient of variation	8.86	8.60	4.45	
TIMP-1 (T-S)	Mean	11.11	14.18	14.30	0.2932 ^A^
	Coefficient of variation	53.46	34.37	40.51	
TIMP-2T	Mean	89.26	91.05	85.39	0.3317 ^A^
	Coefficient of variation	10.15	6.58	7.86	
TIMP-2S	Mean	81.27	78.64	80.18	0.6159^ A^
	Coefficient of variation	10.82	6.83	4.79	
TIMP-2 (T-S)	Mean	8.00	12.42	5.21	0.0085 ^A^
	Coefficient of variation	42.90	43.39	111.16	

N: Nodes, T: Tumor, S: Stroma , A: Analysis of variance, KW: Kruskal-Wallis test, MMP-2: Matrix metalloproteinase-2 MMP-9: Matrix metalloproteinase-9, TIMP-1: Tissue inhibitor of metalloproteinase-1

In the analysis of 3-year and 5-year survivals, a statistical significance for the expression of the MMP-9 (T-S) variable was observed only for 5-year survival. However, it should be emphasized that in the groups of patients who survived in the periods of 3 and 5 years higher levels of MMP-2P, MMP-9N, TIMP-1N, TIMP-1P, TIMP-2N, as well as TIMP-2P, and a lower level of MMP-2N were noticed in comparison to the group of patients who did not survive during the studied periods.

In the multivariate analysis, it was observed that the significantly responsible variables for survival until the third year included MMP-9P and TIMP-1P (P=0.0328, P<0.0343) while until the fifth year they were MMP-2P, MMP-9N, MMP-9P, and TIMP-1P (P=0.0476, P=0.0387, P=<0.0370, and P=0.0337). The odds ratio was calculated and the 95% confidence interval was determined. 

The most significant increase in the expression level was demonstrated for TIMP-1P for 5-year survival [OR=1.724]. Based on the analysis of the estimated odds ratios, it can be concluded that with an increase in MMP-2P, MMP-9N, and TIMP-1P, the chance of survival until the fifth year increased while in the case of MMP-2N, MMP-9P, and TIMP-1N this chance decreased.

## Discussion

The results of several studies, conducted for many years, have demonstrated a correlation between the expression of metalloproteinases and their inhibitors in the process of degradation and reconstruction of the extracellular matrix resulting in the activation of carcinogenetic processes and the formation of metastases in malignant tumors (7-9). The full assessment of this phenomenon requires the analysis of MMPs and TIMPa both within the tumor and its stroma. 

In many publications, the authors also indicated significant correlations between the expression of metalloproteinases and their tissue inhibitor, as well as the survival or onset of disease recurrence. Particular attention in head and neck cancers should be focused on metalloproteinases 2 and 9, which are associated with the invasion by cancer and formation of metastatic lesions. 

Their role is associated with the degradation of type IV collagen and the main component of the extracellular matrix in the head and neck region (7,10-12). The tissue inhibitors of metalloproteinases (TIMP-1 and TIMP-2) play no less important role, and they are the factors influencing the progression of cancerous changes due to interactions with metalloproteinases (7,13).

In numerous reports regarding head and neck cancers, the present correlations are emphasized between the changes in the expression of MMP-2, MMP-9, TIMP-1, and TIMP-2 with tumor progression or its clinical stage (7,10-12). In the study of metalloproteinases 2 and 9 expressions in oropharyngeal cancers, an increase was observed in their expression level depending on the stage and the presence of metastases to lymph nodes (11,14,15). In the above-mentioned studies, expression levels were mainly presented for the tumor tissue while the stroma was ignored. Only previous studies, as well as the studies of Dünne et al., compared the expression levels of MMP-2, MMP-9, TIMP-1, and TIMP-2 in tumor and stromal tissue (10,16). 

The conducted analysis of MMP-2 and MMP-9 expression level in the whole group of patients with oropharyngeal cancer demonstrated a higher expression of MMP-2 in the stroma in comparison to the expression in tumor (P<0.0001), which may indicate the involvement of this metalloproteinase in the processes of tumor progression. In the case of MMP-9, statistically significant lower expression values were observed in stromal tissue (P=0.0445) in comparison to tumor tissue.

Regardless of MMPs, their specific tissue inhibitors, including TIMP-1 and TIMP-2, play an important role in tumor progression. In the available literature concerning oropharyngeal cancers, an increase in TIMP-1 progression was observed in tumors with a higher degree of organ and regional staging. The expression of TIMP-2 did not exhibit a close correlation with the progression of neoplastic lesions (7). 

In the author’s own research, based on the changes in the expression of tissue inhibitors of metalloproteinases in the whole studied group, a higher expression of TIMP-1 and TIMP-2 was observed in tumor tissues, compared to that in the stroma. The expression of TIMP-1 was higher than that of TIMP-2. The difference in the expression of tumor-stroma (T-S) for TIMP-1 (P<0.0001) proved a higher involvement of TIMP-1 in tumor progression in a statistically significant way, which is confirmed by other researchers (7,17).

The results of many studies emphasized the higher expression of metalloproteinase-2 and metalloproteinase-9 leading to the higher invasiveness in various types of cancers, including oropharyngeal cancers (10,11). The findings of numerous studies emphasized the importance of changes in expression levels of MMP-2 and MMP-9 at different stages of cancer of the head and neck region, including organ and regional staging (11,17). 

In cancers of the mouth, tongue, and throat, with the increase of tumor (T) and nodes (N) feature, the expression of MMP-2 and MMP-9 in the tumor, stroma, and serum increased unequivocally demonstrating the involvement of the above-mentioned enzymes in the process of tumor progression (10,17). In this study, a correlation was demonstrated between a higher expression of MMP-2 in tumor, compared to its expression in stroma among patients without metastases to lymph nodes. This finding was also indicated by a statistically significant difference between the expression of MMP-2 in the tumor and stroma (P=0.0038). It was shown that lower values of MMP-2 in tumor occurred in patients with a higher stage of metastasis to cervical lymph nodes (P=0.0150).

The observed decrease in MMP-2 expression in tumor together with the increase of feature N simultaneously existed with the expression increase of MMP-2in the stroma and the expression decrease of TIMP-2 in the stroma, as well as the increase in the tumor (P=0.0085). It can be assumed that a higher expression of MMP-2 in tumor in comparison to stroma indicated a protective role of MMP-2 in relation to the formation of metastases in regional lymph nodes in the initial stage of the disease. 

However, the observation of dynamics and mutual interactions of the studied enzymes leading to increased expression of MMP-2 in stroma from N1+N2 to N3 with a simultaneous exactly inverse decrease in tumor expression confirm the undoubted role of this metalloproteinase in the process of tumor progression. This finding is affirmed in the related literature (17,18). The MMP-2 in the stroma decreased with increasing node size; however, this correlation was not statistically significant, and the expression was significantly higher than in tumor.

A similar expression was observed in relation to MMP-9 in tumor, where its expression was significantly higher in patients without metastases (P=0.0168). It seems that higher expression values of MMP-9 in tumor and stroma for the N1+N2 stage in comparison to the N3 stage are the results of a constant high expression level of TIMP-1, which inhibits the release of metalloproteinase-9. This dependence suggests a lower impact of metalloproteinase-9 on the invasion ability of cancer into the stroma, particularly at a high degree of nodal staging, where the main role is played by the increase in MMP-2 expression. 

It seems that MMP-9 can inhibit the formation of metastases to lymph nodes in oropharyngeal squamous cell carcinoma. The changes in dynamics with increasing N stage indicate a decrease in MMP-9 expression both in tumor and stroma with a simultaneous increase of TIMP-1 expression. The observation of the dynamics of changes in the expression of the studied metalloproteinases indicated an increase in MMP-2 and a decrease in MMP-9 depending on the degree of staging from T2 to T4. This finding demonstrated the involvement of both enzymes with the predominance of MMP-2 in the process of tumor cell proliferation resulting in tumor size. Similar correlations were observed in other studies (6,11).

In the present study, no statistical significance was observed regarding the expression of TIMP-1 and TIMP-2 in tumor and stroma depending on the T stage. However, based on the observation of the dynamics of changes depending on the T stage, significantly higher values of TIMP-1 expression were registered in tumor stroma (P=0.0454). Similar trends were observed in a study conducted by Burduk et al. (16). In the assessment of nodal staging from N0 to N3, the expression of TIMP-1 and TIMP-2 was higher in tumor tissues, compared to that in the stroma (P=0.2932, P=0.0085). Higher expression of TIMP in tumor may have an initiating role in the process of tumor invasion (7).

In the case of high expression of MMP-2 and MMP-9, both in tumor and its stroma, lower survival rates were demonstrated for squamous cell carcinomas of the tongue and digestive tract (10). In only two available publications concerning throat cancers, higher survival rates were observed in patients with metastases to lymph nodes, as well as higher expression of MMP-9, and no effect of higher expression of TIMP-1 on the analyzed survivals (15).

In the present study, no correlation was observed between the expression of MMPs and TIMPs with 3-year and 5-year survivals. Despite the lack of significance, a higher expression of MMP-2 in stroma and tumor was observed in patients who survived in 3 and 5 years, and an inverse correlation for the expression of MMP-9, which was confirmed based on the literature. Only the introduction of the MMP-9 variable or expression difference for metalloproteinase in tumor and stroma allows for the assessment of prognoses concerning the survival of the patient. This result gives a certain possibility of using MMP-9 (T-S) in the diagnosis of patients with oropharyngeal squamous cell carcinoma. 

In contrast to the exclusive metalloproteinases levels, this factor can be helpful in the assessment of the prognoses concerning 5-year survival (P=0.0299). In the case of the analysis of TIMP-1 and TIMP -2 expressions, in both cases, a higher expression of inhibitors in tumor and stroma was noticed in patients who survived in 3 and 5 years; however, no statistical significance was observed.

Following the obtained results of the multivariate analysis, it was noted that three parameters, including MMP-9P, MMP-9N, and TIMP-1P were helpful in the evaluation of 3-year survival. However, in the analysis of 5-year survival, MMP-2 and MMP-9 both in tumor and stroma, as well as TIMP-1 in stroma, can be useful. It was concluded that the increase in MMP-2, TIMP-1 in tumor, and MMP-9 in stroma were characterized by a decrease in the odds ratio for 5-year survival. 

An increase in the predictive power of the mutual complex interaction within the metalloproteinases system can play an important role in the diagnosis of the patient. A significant increase in the odds ratio for survival occurred in the case of increased expression of MMP-9 investigated in the tumor and MMP-2 and TIMP-1 in the stroma; however, the chance of the patient’s survival increased most clearly in relation to TIMP-1P [OR=1.724].

## Conclusion

In oropharyngeal cancer dynamic changes, the expression of metalloproteinases and their inhibitors occurred both in the tumor and stroma. The increased expression of MMP-2 and MMP-9 within tumor decreases the risk of metastases to cervical lymph nodes in the initial stage of the disease. In turn, the increase of MMP-2 expression in stroma and its decrease in tumor occurs with the increasing degrees of regional staging; however, MMP-9 expression decreases in both locations with the increase of the N feature. 

In the multivariate analysis, the factors which influence 3-year survival included the changes in MMP-9 expression in the tumor and stroma, as well as TIMP-1 in the stroma. Additionally, 5-year survivals were influenced by the changes in the expression of MMP-2 investigated in both locations.
